# Synthesis of Magnetic Core–Shell Materials and Their Application in Detection of Food Contaminants

**DOI:** 10.3390/foods14193305

**Published:** 2025-09-24

**Authors:** Jing Cao, Huilin Li, Jingjing Cui, Mengmeng Gao, Jingming Sun, Mingfei Pan

**Affiliations:** 1 Key Laboratory of Food Quality and Health of Tianjin, Tianjin University of Science and Technology, Tianjin 300457, China; cao139327@163.com (J.C.); lhltust@163.com (H.L.); cjj0040@163.com (J.C.); gaomeng200123@163.com (M.G.); 03017@qqhru.edu.cn (J.S.); 2 State Key Laboratory of Food Nutrition and Safety, Tianjin University of Science & Technology, Tianjin 300457, China

**Keywords:** magnetic core–shell nanoparticles, food contaminants, synthesis, application

## Abstract

Food contamination poses a significant global public health challenge, necessitating the accurate detection of hazardous substances within complex food matrices. Magnetic core–shell nanomaterials have emerged as critical materials for trace contaminant analysis due to their efficient magnetic separation capabilities, excellent adsorption performance, and tunable surface functionalities. By encapsulating magnetic cores with functional shells, these nanomaterials combine rapid magnetic responsiveness with advantageous shell properties, including target-specific recognition, enhanced dispersibility, colloidal stability, and high surface area. This enables a comprehensive detection approach encompassing target adsorption, rapid separation, and signal amplification. Magnetic core–shell nanomaterials have been effectively integrated with techniques including magnetic solid-phase extraction (MSPE), fluorescence (FL) assays, and lateral flow immunoassays (LFIAs), demonstrating broad applicability in food safety monitoring and detection. This review outlines synthesis strategies for magnetic core–shell nanomaterials, highlights their applications for food contaminant detection, and discusses future challenges and prospects in the field of food safety analysis.

## 1. Introduction

Food safety continues to represent a critical global public health issue, with an estimated 600 million cases of foodborne illness occurring annually, highlighting the urgent need for accurate detection of contaminants [1,2,3,4]. Current challenges for this process are primarily attributed to the trace levels of contaminants, the complex composition of food matrices, and the bioaccumulation potential of various substances within the food chain [5,6,7,8]. Although conventional methods such as chromatography, mass spectrometry, and surface-enhanced Raman spectroscopy (SERS) offer reliable detection capabilities, their effectiveness is frequently hindered by matrix interference, which necessitates effective sample pretreatment [9,10,11,12]. Traditional extraction techniques, including solid-phase extraction (SPE) and liquid–liquid extraction, are inherently plagued by critical limitations: excessive solvent consumption, time-intensive protocols, and inadequate selectivity [13,14,15]. These limitations become especially pronounced when pretreatment and detection systems are not integrated, leading to issues such as signal instability due to matrix effects [16].

This technological gap has driven the development of MSPE as a sustainable alternative. MSPE leverages the unique properties of magnetic nanomaterials to achieve high-throughput enrichment of contaminants [13,17,18]. The efficacy of MSPE is fundamentally determined by the structural design of the magnetic adsorption materials. Magnetic nanomaterials (MNMs), recognized as superior magnetic adsorbents, exhibit favorable attributes, such as a large specific surface area, excellent magnetic responsiveness, facile functionalization, ease of separation, non-toxicity, and reusability. These characteristics establish MNMs as the core adsorption carriers in MSPE [19,20,21]. In complex food matrices, components like high salt, high protein, and multiple impurities impose stringent requirements on the stability and targeting ability of materials for contaminant detection and removal. Compared with traditional nanomaterials with a single structure, magnetic core–shell materials exhibit significant advantages in practical food matrices. One of the core reasons lies in the unique protective and shielding effect of the shell layer. Although Fe_3_O_4_ is prone to oxidation by air (which leads to the loss of magnetic properties), the shell layer can protect the magnetic core from external erosion through physical isolation, thus enabling the material to maintain stability in complex food matrices [22,23]. For instance, a dense SiO_2_ shell layer can act as a barrier to prevent H^+^ in acidic environments from directly contacting the magnetic core, thereby improving the overall magnetic stability of the material. Beyond the aforementioned effect, certain shell layers (such as metal–organic framework (MOFs) and molecularly imprinted polymers (MIPs)) can further enhance the targeted adsorption of analytes through their inherent sites. This is precisely the second reason why magnetic core–shell materials outperform traditional nanomaterials in the practical detection of food matrices. Moreover, the shell layer can shield macromolecular impurities in food through pore-size screening or surface modification, reducing the occupation of functional sites on the material and ensuring the efficiency of the material’s targeted action toward target contaminants [24,25]. Functional shell layers can be engineered on magnetic nanoparticles (MNPs) to form core–shell architectures—for example, by grafting specific functional groups [26], incorporating MIPs [27], or immobilizing nanobodies [28]. These materials can efficiently concentrate harmful substances within complex food matrices and significantly improve the detection sensitivity of trace pollutants [29].

More importantly, the functional integration of magnetic core–shell structures goes beyond the traditional role of pretreatment materials: the magnetic core enables rapid separation, while the functionalized shell (such as those loaded with SERS-active substrates, fluorescent probes, or immune recognition units) directly enhances signal amplification. Consequently, this design can be seamlessly integrated with advanced analytical technologies, including quantum-dot-based cooperative sensors, aptamer biosensors, LFIAs, and SERS, thereby achieving the integration of “enrichment–separation–detection” [28,30,31,32,33]. This integrated approach offers a more effective solution to challenges such as matrix interference and cumbersome operational procedures commonly encountered in traditional detection methods [34].

Although MNPs are the foundational component of core–shell structures, they still have limitations: inherent instability, a tendency to aggregate, low target specificity, and limited potential for surface modification [35]. The development of magnetic core–shell nanomaterials has effectively addressed these challenges through innovative design of functional shells. When magnetite (Fe_3_O_4_) nanoparticle cores are encapsulated with precisely engineered shell materials, the resulting composite structures exhibit precisely tunable properties tailored for specific applications [19]. The functional shell significantly enhances MNPs by providing three key improvements: adjustable interfacial properties, selective adsorption capabilities, and significantly improved dispersion stability [24]. Different shell materials impart distinct functionalities—SiO_2_ or glutathione (GSH) coatings enhance colloidal stability and prevent nanoparticle aggregation through controlled shell thickness [36,37]; bio-recognition shells incorporating aptamers, antibodies, or MIPs provide exceptional molecular specificity [38,39,40]; and MOF coatings offer the combined advantages of ultrahigh surface area, tailored porosity, and accessible metal active sites [41]. This synergistic integration of magnetic core and functional shell results in nanomaterials with superior performance in complex food matrices, transforming modern approaches to food safety detection [42]. Therefore, this review provides a comprehensive examination of the various synthesis strategies currently employed in preparing magnetic core–shell nanomaterials for food hazard detection. It systematically summarizes their applications throughout the entire detection process and outlines future development directions for these nanomaterials in the field of food analysis.

## 2. Synthesis Strategies of Magnetic Core–Shell Nanomaterials Composite

The synthesis of magnetic core–shell materials poses considerable challenges owing to the complexity of multi-step preparation procedures and the necessity for concurrent reaction conditions. The ultimate performance is critically dependent on the chosen synthesis strategy [43,44]. To address this, researchers have proposed a range of fabrication techniques suitable for food analysis applications, such as coprecipitation, in situ synthesis, chemical deposition, self-assembly, and sacrificial template methods. These methodologies enable the production of diverse magnetic core–shell composites with tailored properties (Table 1).

### 2.1. Coprecipitation Method

The coprecipitation method is a prominent technique for fabricating magnetic core–shell materials for food detection applications, offering notable advantages including environmental sustainability and cost-effectiveness [45]. This approach involves the simultaneous precipitation of multiple ionic species through alkaline reagent addition under controlled temperature conditions, typically performed in an inert atmosphere [46]. Research demonstrates that precise regulation of nucleation kinetics and crystal growth rates enables the fabrication of nanoparticles featuring narrow size distributions [47]. Critical parameters such as pH and temperature can be systematically adjusted to control product morphology and crystallinity, allowing structural customization for diverse food matrices—particularly challenging samples with high protein or fat content.

Magnetic core–shell nanomaterial synthesis typically starts with the preparation of the magnetic core component, most commonly magnetite nanoparticles (Fe_3_O_4_). Although the traditional coprecipitation approach stands as the most commonly utilized technique for the synthesis of Fe_3_O_4_, it often yields particles with limited crystallinity and magnetic properties. To address these limitations, Zhang et al. developed a hybrid heating–cooling coprecipitation technique (HMIHC), which incorporated alternating magnetic fields with controlled alkali addition [45]. This innovation enabled precise phase control during the synthesis process, leading to Fe_3_O_4_ nanoparticles with enhanced magnetization due to the improved alignment of the internal magnetic moment. Consequently, the synthesized materials exhibit superior magnetic responsiveness, significantly enhancing analyte separation efficiency in complex food samples. Alternatively, Omelyanchik et al. proposed a glycine-assisted one-step coprecipitation method that enables simultaneous size regulation and surface functionalization [48]. All glycine-functionalized iron oxide MNPs produced through this method displayed room-temperature superparamagnetism, which prevented particle aggregation while ensuring stable dispersion and improved adsorption performance in complex food matrices.

**Figure 1:**
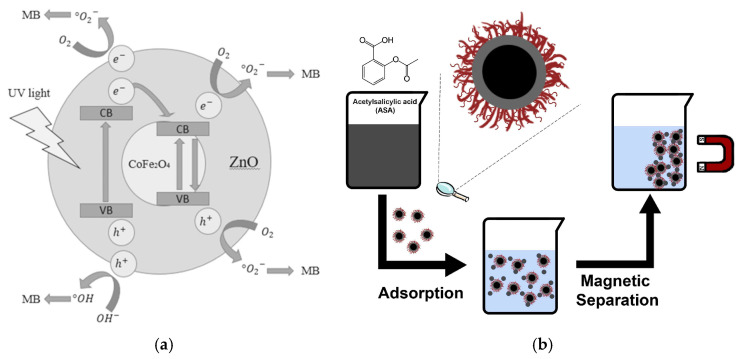
(**a**) Schematic of the magnetic core–shell CoFe_2_O_4_@ZnO nanoparticles [49]. Copyright Materials Research Express, 2020. (**b**) Schematic diagram of the preparation of CoFe_2_O_4_-γ-Fe_2_O_3_-Lys core–shell nanoparticles [51]. Copyright Nanomaterials, 2023.

In the case of coating magnetic cores to synthesize magnetic core–shell nanomaterials, the coprecipitation method presents notable advantages. Suharyadi et al. synthesized CoFe_2_O_4_@ZnO core–shell nanoparticles using this technique. The resulting CoFe_2_O_4_/ZnO heterojunction significantly improved the stability of the material, enabling repeated applications in food analyses including multiple enrichment and detection cycles. This material demonstrated a methylene blue degradation efficiency of 78.3%, highlighting its remarkable catalytic capability (Figure 1a) [49]. Notably, the size distribution of MNPs plays a critical role in its functionality: broad size distributions tend to compromise magnetic properties, whereas uniform nanoparticles enhance both magnetization and the reproducibility of ultra-trace analyte detection. During the coprecipitation procedure, alkaline conditions fundamentally influence nanoparticle size. Stronger alkaline environments generally lead to larger particles. [50]. For instance, Maciel et al. synthesized size-tunable CoFe_2_O_4_-γ-Fe_2_O_3_-Lys core–shell nanoparticles with average diameters of 8.5 nm and 13.5 nm by adjusting the pH. The optimized material demonstrated a high aspirin adsorption capacity of 16.4 mg/g, confirming the size-dependent behavior of these nanomaterials in the analysis of residues (Figure 1b) [51].

As a versatile synthesis technique, the coprecipitation method allows for precise control over nanoparticle size and magnetic characteristics by modulating key parameters such as Fe^2+^/Fe^3+^ molar ratio, pH, temperature, and stirring rate [52]. Although the optimization of these parameters requires careful coordination, well-established protocols can ensure the reproducible synthesis of uniform nanoparticles. This consistency is essential for developing standardized food detection methods that require uniform performance across different production batches.

### 2.2. In Situ Synthesis Method

The in situ growth method represents an innovative approach for synthesizing core–shell nanomaterials by means of the bottom-up chemical reduction of metal salts. This method induces controlled nucleation and gradual shell layer formation on substrate surfaces. By precisely regulating core dimensions and shell thickness, this method enhances the detection sensitivity of trace substances—critical for identifying minute food contaminants [46,53]. Earlier studies by Morel et al[54]. demonstrated the synthesis of monodisperse and non-aggregated Fe_3_O_4_@SiO_2_ nanoparticles using sonochemical techniques. Ultrasonic treatment facilitated the formation of a uniform silica shell around the core, achieving nanoscale precision in shell thickness. The resulting uniform core–shell structure effectively minimized material aggregation in complex food matrices, such as fruit juices and emulsions, thereby facilitating subsequent separation processes. Beyond its capacity for precise size control, the in situ growth method also offered considerable process flexibility, enabling the tailored design of nanostructured materials. This characteristic provided a distinct advantage in the field of food safety testing, particularly in applications requiring specific interfacial properties.

#### 2.2.1. Preparation of Magnetic Core–Shell Materials with MOF Shells

**Figure 2:**
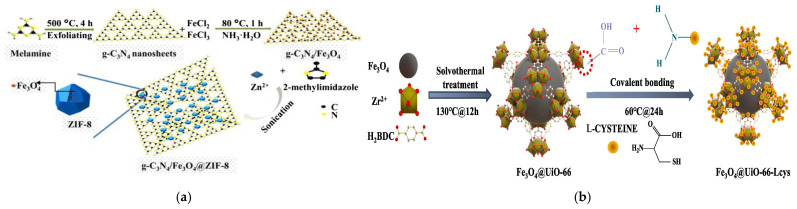
(**a**) Diagrammatic illustration of the in situ preparation of g-C_3_N_4_/Fe_3_O_4_@ZIF-8 nanocomposites [56]. Copyright Microchimica Acta, 2020. (**b**) Synthesis process of Fe_3_O_4_@UiO-66-Lcys [57]. Copyright Chemical Engineering Journal, 2024.

In situ synthesis represents a crucial approach for fabricating magnetic core–shell composites with MOF shells. Sharma et al. demonstrated the application potential of MOF shells in broad-spectrum capture of pathogenic bacteria: they successfully synthesized Fe_3_O_4_@SiO_2_@NH_2_-MIL-125(Ti) nanocomposites through solvothermal grafting of NH_2_-MIL-125(Ti) shells onto Fe_3_O_4_@SiO_2_ cores [55]. For this type of material (SiO_2_ shells and MOF shells), SiO_2_ shells can not only effectively prevent the agglomeration of Fe_3_O_4_ particles but also prevent their oxidation. The mesoporous structure of the MOF shell of this material only allows bacteria to enter the internal active sites, thus restricting the interference from macromolecular substances in the matrix with the adsorption process. In addition, the pores of the MOF exhibit hydrophobic interactions, which can synergize with the electrostatic adsorption between the material and bacteria to further enhance the adsorption effect. The synthesis process of this material only requires the hydrothermal method, without the need for additional high-temperature or high-pressure conditions. In a single adsorption operation, the material dosage is only 1 mg/mL, and the overall process is simple with low energy consumption, providing favorable conditions for industrial applications. Additionally, the semi-core–shell structured material g-C_3_N_4_/Fe_3_O_4_@ZIF-8 developed by Qi et al. also possesses the capability of broad-spectrum capture, and this material enables the simultaneous extraction of 15 sulfonylurea herbicides when coupled with MSPE-LC-MS/MS (Figure 2a) [56]. Li et al. developed the Fe3O4@UiO-66-Lcys material, which has both high yield and strong adsorption capacity, with an adsorption capacity of up to 430.89 mg/g for Cd^2+^ (Figure 2b) [57]. Using the same framework, Wang et al. synthesized the Fe_3_O_4_@SiO_2_@UiO-66-NH_2_ composite. When combined with high-performance liquid chromatography (HPLC) analysis, this composite achieved recoveries of 95.83–101.5% for ochratoxin A across three concentration levels and a remarkable detection limit of 0.3 μg/kg [58]. These materials exhibit excellent performance due to the inherent structural characteristics of MOFs—high specific surface area, tunable pore structure, accessible metal active sites, and modifiable surface chemistry. These features work synergistically to enhance contaminant enrichment efficiency [57].

#### 2.2.2. Preparation of Magnetic Core–Shell Materials with MIP Shells

MIPs exhibit excellent physicochemical stability, high target specificity, and reusability. When combined with MNPs to form magnetic molecularly imprinted nanoparticles (MMINs), these composite materials significantly improve the extraction selectivity while reducing the matrix interference effects [59]. As a representative core–shell architecture prepared via in situ synthesis, such materials offer distinct advantages for the precise enrichment and detection of trace contaminants.

**Figure 3:**
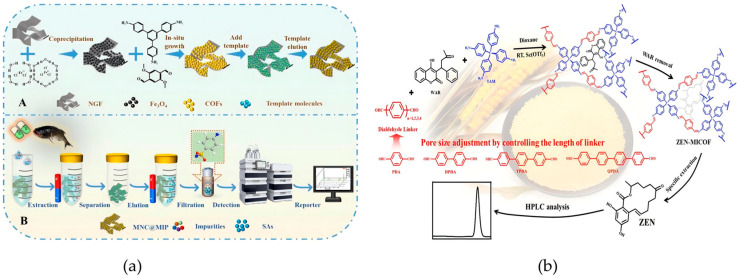
(**a**) Schematic for the synthesis of MNC@MIPs (A) and MNC@MIPs-based MSPE combined with HPLC for SAs detection (B) [60]. Copyright Food Chemistry, 2024. (**b**) Schematic illustration of pore-size regulation strategies for ambient-temperature synthesis of MICOF nanospheres in selective extraction of zearalenone from cereal samples [61]. Copyright Analytical Chemistry, 2024.

Sun et al. took the lead in constructing the core structure of the “magnetic substrate COFs carrier-MIPs recognition layer”, in which COFs were synthesized in situ on magnetic nitrogen-doped graphene foam (MNGF), followed by molecular imprinting to produce MNC@MIPs (Figure 3a) [60]. The in situ grown COFs enable small-molecule sulfonamide drugs to be screened out while blocking interference from macromolecules such as proteins in fish matrices. On the other hand, MIPs form specific recognition cavities through the pre-assembly of template molecules, which only allow sulfonamide drugs to pass through matching binding, thus excluding structural analogs. This material is prepared via an in situ method and free radical polymerization reaction, without the need for high-temperature and high-pressure conditions. A single adsorption cycle requires only 15 mg of the material, and the adsorption rate remains 87% after 10 cycles. In addition, the regeneration process only requires ultrasonic cleaning, which significantly reduces the cost per use. In the design of molecularly imprinted materials, pore size is a key parameter influencing adsorption capacity and selectivity: building upon this approach, Li et al. proposed a room-temperature pore-size regulation strategy for molecularly imprinted COFs (MICOFs) targeting zearalenone (ZEN) (Figure 3b) [61]. Utilizing 4-aminophenyl as a functional monomer and size-tunable dialdehyde monomers, the system achieved a maximum ZEN adsorption capacity of 177.2 mg/g and an imprinting factor of 10.1. This approach effectively addressed the selectivity limitations commonly associated with conventional COFs in the extraction of mycotoxins from cereal matrices.

MIPs have emerged as powerful functional materials for specific recognition in analytical applications, and their performance—particularly in selectivity, sensitivity, and adaptability to complex matrices—largely hinges on structural design innovations. Beyond traditional core–shell architectures, recent research has expanded into novel morphologies such as rod-like structures, which have shown enhanced analytical performance. Guadaño et al. developed halloysite nanotube-core MIPs with magnetite functionalization, demonstrating remarkable selectivity for cyclopiazonic acid and recoveries of 83–97% in rice flour, with an LOD of 11.1 μg/kg and LOQ of 36.5 μg/kg [62]. Similarly, Khojasteh et al. fabricated multi-walled carbon nanotube (MCNT)-based rod-like MIPs, achieving a detection limit of 1.3 μg/L for fenitrothion [63]. In addition, Gao et al. reported that their MCNT@MIPs nanocomposites could detect sulfonamide antibiotics at a concentration as low as 0.1 μg/kg [64]. These advancements illustrated how innovative structural designs can overcome the limitations of conventional materials, thereby enabling highly sensitive multi-residue detection in complex food matrices.

#### 2.2.3. Preparation of Magnetic Core–Shell Materials with Magnetic MOF and Molecularly Imprinted Multilayer Shells

MIPs have remarkable molecular recognition capabilities, while magnetic MOFs have superior adsorption performance. By strategically integrating the porous structure of MOF as an intermediate layer with the surface imprinting technology of MIPs, a functional system is formed, combining magnetic separation, high-efficiency adsorption, and specific molecular recognition. This synergistic approach effectively meets the key requirement for concurrent concentration and identification of ultra-trace contaminants in intricate food matrices.

**Figure 4:**
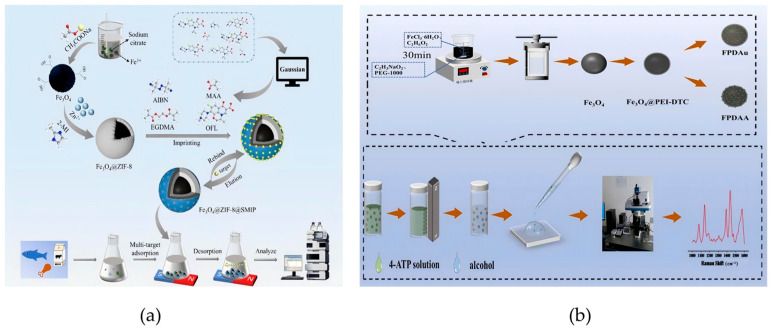
(**a**) Schematic diagram of the fabrication process for Fe_3_O_4_@ZIF-8@SMIP used in FQ detection [65]. Copyright Food Chemistry, 2024. (**b**) Schematic illustration of the lab-based fabrication of FPDAA, along with the selection and detection procedures for molecular probe tests [71]. Copyright Journal of Alloys and Compounds, 2024.

Our previous work demonstrated the successful fabrication of Fe_3_O_4_@ZIF-8@SMIP multilayer core–shell composites through an in situ growth method (Figure 4a) [65]. This material exhibited remarkable adsorption capacities from 88.61 to 212.93 mg/g and achieved rapid equilibrium within 2–3 min for various fluoroquinolone antibiotics. This approach overcame the limitations of conventional materials in detecting multiple antibiotic residues in animal-derived foods, particularly with respect to matrix interference and enrichment efficiency. Compared with traditional SPE materials, this shortens the pretreatment time of a single sample to less than one-fifth, laying a foundation for the batch processing of multiple samples. For another type of food safety risk—the detection of bisphenol A (BPA) migration from food contact materials—Zhu et al. developed an efficient and environmentally benign synthesis strategy for magnetic copper-based multifunctional molecularly imprinted polymers (MCMMIPs) [66]. The resulting materials displayed superior magnetic properties (20 emu/g), high surface area (289.4 m^2^/g), and distinctly structured imprinted sites, enabling the sensitive detection of BPA migration from food contact materials, with a detection limit of 1.01 μg/kg and recoveries of 72.1–102.85%.

These findings collectively demonstrate that magnetic MOF-MIP hybrid nanomaterials exhibit advantages such as rapid mass transfer, high binding capacity, and uniformly distributed recognition sites [67]. Due to these distinctive properties, they are particularly effective in the process of detecting trace-level contaminants within complex food matrices, thereby presenting substantial potential for applications in food safety.

#### 2.2.4. Preparation of Magnetic Core–Shell Materials with Noble Metal Shells

Precious metals (Au, Ag) demonstrate exceptional SERS activity through surface plasmon resonance effects. By employing in situ reduction methods (such as sodium citrate-mediated reactions), precisely controlled precious metal shells can be grown on magnetic cores to create integrated “magnetic separation–signal enhancement” core–shell structures. These hybrid nanocomposites combine SERS enhancement with magnetic responsiveness, enabling multi-parameter nondestructive quantitative detection, making them ideal platforms for ultrasensitive food contaminant analysis [68,69,70]. Researchers have explored two approaches—adjusting noble metal shell thickness and designing noble metal core structures—to provide targeted solutions for detecting different food contaminants.

Liu et al. developed Fe_3_O_4_@PEI-DTC-Au@Ag composites with tunable Ag shell thickness through in situ synthesis. Using 4-aminothiophenol (4-ATP) as a probe, the best-suited substrate (Fe_3_O_4_@PEI-DTC-Au@Ag-3) achieved an impressive enhancement factor of 1.121 × 10^6^ (Figure 4b) [71], enabling trace-level thiram detection while simplifying traditional pretreatment steps into a single enrichment–detection process. In this material, the PEI-DTC interlayer can “anchor” Au@Ag nanoparticles through electrostatic interactions. The gaps in the Au@Ag shell form nanoscale hotspots, which only allow small-molecule thiophanate to enter while blocking macromolecular interference. In terms of the mechanism, the “magnetoplasmonic effect” of the Fe_3_O_4_ magnetic core and the surface plasmon resonance effect of the Au@Ag shell act synergistically, thereby significantly enhancing the electric field intensity of FPDAA-3. Meanwhile, the PEI-DTC layer can enrich the target thiophanate through electrostatic attraction, and Au@Ag can enhance adsorption with the hydrophobic groups of thiophanate via hydrophobic interactions. In terms of preparation, the material only requires the hydrothermal method and seed deposition method, without the need for high-temperature and high-pressure equipment; the entire process can be completed using conventional laboratory instruments. For application, only 1 mg of the material is needed for a single SERS detection. After fivecycles, the SERS signal of thiophanate can still be maintained at >90%, thus reducing the generation of solid waste. Overall, it features low cost and is easy to apply to industrial production. In the structural design of noble metal components, Hoang et al. broke through the conventional approach of “using noble metals as the shell layer” and synthesized size-tunable Ag@Fe_3_O_4_ core–shell nanoparticles via solvothermal in situ synthesis. Using methylene blue as a probe, these nanoparticles showed excellent SERS performance with high reproducibility, establishing a reliable approach for rapid detection of illegal food additives [68]. The study highlighted how precise control over shell synthesis and post-treatment parameters can optimize sensor performance.

#### 2.2.5. Preparation of Magnetic Core–Shell Materials with Functional Polymer Shells

Functional polymers, including polydopamine (PDA) and aptamer-functionalized polymers, have become preferred materials for constructing functional shells in magnetic core–shell composites due to their structural tunability, facile modification, and superior biocompatibility. The strategic combination of these polymer-modified magnetic nanomaterials with SERS technology offers an innovative technique for quick detection of trace contaminants in food matrices.

**Figure 5:**
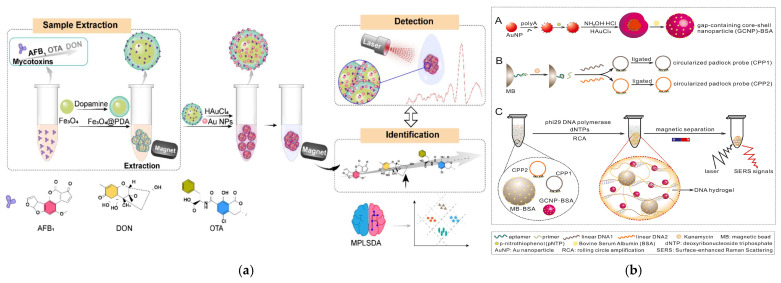
(**a**) A diagram illustrating the working mechanism of the SERS-based nanosensor for mycotoxins detection [72]. Copyright Food Chemistry, 2024. (**b**) Schematic illustration of the SERS aptasensor based on DNA hydrogel fishing strategy. (A) Synthetic procedures of GCNPs. (B) Competitive binding assay for KANA and formation of circularized padlock probe. (C) DNA hydrogel formation through RCA reaction and SERS detection. [73]. Copyright Biosensors and Bioelectronics, 2022.

Different research teams have generated precise designs targeting different food matrices and contaminant types. Zhang et al. developed Fe_3_O_4_@PDA composites through dopamine oxidative self-polymerization on Fe_3_O_4_ cores (Figure 5a) [72]. Subsequent deposition of SERS-active Au nanoparticles created a platform capable of rapid mycotoxin enrichment in cereals and nuts without extensive pretreatment, achieving a notable detection limit of 1 ng/mL. After the Fe_3_O_4_ core is coated with a PDA shell, the material can adsorb mycotoxins via π-π stacking and hydrogen bonds from -OH/-NH_2_ groups, thus preventing interference from macromolecules in corn matrices. The Au nanoparticles attached to the material surface can function as the metal shell mentioned earlier, and also block the entry of macromolecular impurities. This material is synthesized via a two-step method, combining the hydrothermal method with room-temperature seed deposition, and has relatively low cost. In response to the higher sensitivity requirements for the detection of antibiotic residues in animal-derived foods, Chen et al. engineered MBs@aptamer core–shell structures using magnetic beads as cores, which, when combined with SERS primers, enabled ultrasensitive kanamycin detection at 2.3 fM concentrations, demonstrating exceptional potential for monitoring antibiotic residues in animal-derived products (Figure 5b) [73].

Despite the operational simplicity of in situ synthesis for preparing magnetic core–shell nanomaterials, several critical challenges remain. The method demands stringent control over reaction system compatibility and synthesis conditions, while requiring deeper mechanistic understanding. A primary technical hurdle involves potential incorporation of reaction-medium impurities at core–shell interfaces, which may compromise detection stability [74]. Current research often addresses this limitation by combining in situ synthesis with complementary techniques to optimize composite performance.

### 2.3. Alternative Synthesis Strategies for Magnetic Core–Shell Materials

Beyond coprecipitation and in situ synthesis approaches, alternative fabrication strategies including physical coating and chemical vapor deposition (CVD) have emerged as valuable methods for producing magnetic core–shell materials tailored to food detection applications. These techniques enable precise control over core–shell interface characteristics and facilitate specialized structural designs critical for analytical performance.

#### 2.3.1. Physical Coating Method

Physical coating methods rely on encapsulating core materials within shell matrices through non-chemical processes such as thermal melting, casting, and mechanical confinement, without forming chemical bonds or involving complex reactions. This physical encapsulation approach makes them particularly suitable for developing low-toxicity core–shell materials intended for direct food contact applications.

**Figure 6:**
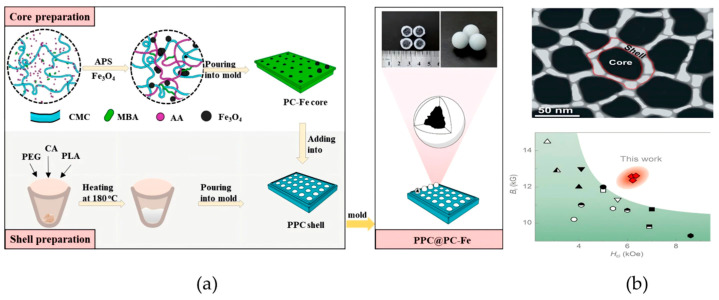
(**a**) Schematic of the preparation process for PPC@PC-Fe core–shell hydrogel [75]. Copyright Carbohydrate Polymers, 2024. (**b**) Characterization of core/shell-like Nd_2_Fe_14_B/α-Fe and comparison of the magnetic properties of nanocomposite magnets [76]. Copyright Nano Letters, 2016.

With the goal of “reducing heavy metal content in food systems”, Hu et al., fabricated magnetic core–shell spheres by casting a molten mixture of polylactic acid (PLA), polyethylene glycol (PEG), and citric acid (CA) at 180 °C to form a polymeric shell (PPC) around PPC@PC-Fe cores, where Fe_3_O_4_ nanoparticles were embedded in a polyacrylic acid/carboxymethyl chitosan (PAA/CMC) hydrogel network (Figure 6a) [75]. This study quantitatively determined that the adsorbent’s production cost is USD 7.31 × 10^3^ per metric ton, much lower than that of traditional adsorbents (e.g., activated carbon). The shell components (PLA, PEG, CA) are all FDA-certified food-grade and pose no health hazards. The release amount of Fe^3+^ from the core was only 7.53% within 10 days, and the magnetic recovery rate in aqueous solution reached 97%, which prevents residue in the environment. This material can be prepared using simple molds without complex processes, making it suitable for mass production, and is particularly suitable for matching the pH of farmland soils and remediation cycles. Li et al. focused on the need to “improve magnetic separation efficiency in food analysis”. They developed Nd_2_Fe_14_B/α-Fe nanocomposite ribbons via rapid quenching, forming a core–shell structure with the hard-magnetic Nd_2_Fe_14_B phase as the core and soft-magnetic α-Fe as the shell (Figure 6b) [76]. The nanocrystalline growth during melt processing yielded simultaneous high remanence and coercivity, enhancing magnetic separation efficiency in food analysis.

While physical coating alone remains less common for magnetic nanomaterial synthesis due to weaker core–shell adhesion, it is frequently combined with other techniques to improve stability while maintaining detection performance.

#### 2.3.2. Chemical Vapor Deposition

CVD constitutes a vacuum deposition method that relies on chemical reactions of gaseous reagents to produce materials. Typically, this technique includes exposing a substrate to gaseous precursors and solid substances (thin films or nanoparticles). The gaseous precursors then react with the solid materials to generate volatile by-products, which are subsequently extracted from the reactor by a pump [43]. CVD offers numerous advantages, such as the elimination of solvent use and easier formation of uniform shell structures [77]. This provides a synthesis strategy for high-reproducibility materials required for identifying ultra-trace pollutants in foodstuffs.

Recent advancements have expanded CVD applications for magnetic core–shell nanomaterials. The aerosol-assisted CVD (AACVD) approach enables rapid (30–60 s) synthesis of spherical Fe_3_O_4_@C nanocomposites while allowing precise control over core–shell dimensions and iron oxide content [78]. This rapid and controllable synthesis characteristic facilitates the mass production of materials for food detection and ensures the reproducibility of detection methods. Additionally, metal–organic CVD (MOCVD) has proven effective for preparing MnFe-C core–shell nanoparticles with adjustable metal proportions, demonstrating exceptional microwave absorption capabilities [79]. Innovative hybrid methods combining CVD with other techniques have shown promise. For instance, spray-drying-assisted CVD using iron nitrate/silica precursors successfully produced multilayer graphene-coated Fe/Fe_2_O_3_@C core–shell nanostructures [80].

This method shows potential in constructing electrochemical sensors for food hazards (such as detecting pesticide residues and illegal additives). CVD, while advantageous for synthesizing magnetic core–shell nanomaterials, imposes specific requirements for experimental setup. The technique necessitates specialized equipment such as vacuum systems, high-temperature furnaces, and precision gas flow controllers, significantly increasing synthesis costs. Additionally, CVD is primarily limited to inorganic shell materials like metals, metal oxides, and carbon, with limited applicability for polymer coatings. Furthermore, the relatively low production efficiency of CVD restricts its potential for large-scale manufacturing. These drawbacks restrict its application in food detection scenarios requiring biofunctionalized shells (such as antibody-modified ones).

#### 2.3.3. Self-Assembly Technology

Molecular self-assembly technology enables the ordered organization of nanostructures through intermolecular interactions, primarily driven by electrostatic forces or specific chemical bonding between molecules and substrates. This bottom-up approach facilitates precise control over shell composition, thickness, and structural organization at the nanoscale [81], offering a versatile platform for developing multifunctional core–shell materials that combine magnetic responsiveness, molecular recognition, and signal transduction capabilities in applications related to food safety. To address multiple needs in food detection, different research teams have applied self-assembly technology to develop magnetic nanomaterials suitable for various scenarios.

**Figure 7:**
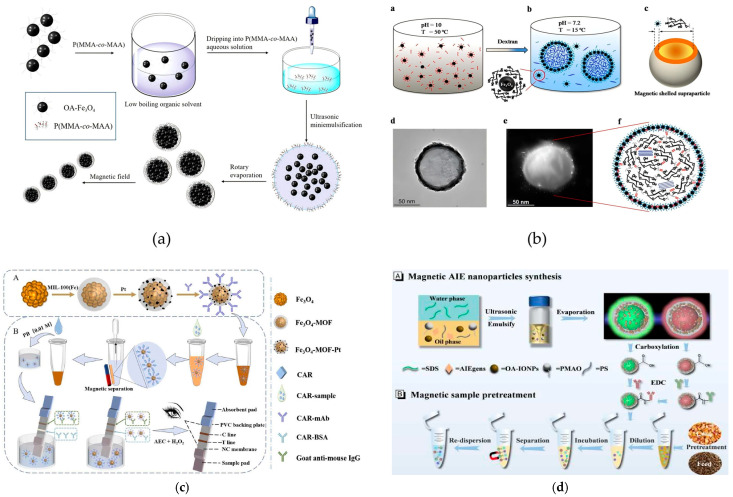
(**a**) Sketched schematic of the self-assembly of Fe_3_O_4_@(PMMA-co-MAA) nanoparticles via amphiphilic copolymer [82]. Copyright *ACS* Applied Nano Materials, 2020. (**b**) Sketched schematic of the self-assembly process for GgSNP shelled core/shell structures (a–f) [83]. Copyright ACS Applied Materials & Interfaces, 2016. (**c**) Schematic representation of a multifunctional Fe_3_O_4_-MOF-Pt-based CAR for LFIA detection. (A) Scheme of antibody-modified Fe_3_O_4_-MOF-Pt preparation. (B) Mechanism of LFIA detection [84]. Copyright Journal of Hazardous Materials, 2024 (**d**) (A) Schematic representation of the synthesis route for MANP via a microemulsion method. (B) Magnetic pretreatment of feed and corn matrices using the MANP probes [85]. Copyright Food Chemistry, 2025.

Focusing on the need for “rapid separation in complex food matrices”, Xu et al. proposed a rapid self-assembly process to synthesize polymer-coated Fe_3_O_4_ magnetic colloidal nanoparticles (MCNPs) within 30 min (Figure 7a) [82]. The resulting nanoparticles showed excellent dispersibility, high Fe_3_O_4_ content (84.48 wt%), and strong saturation magnetization (69.96 emu/g), making them ideal for efficient magnetic separation in complex food matrices. Zheng et al. developed supercooled self-assembled magnetic shell nanomaterials using glucose as a core material (Figure 7b) [83]. This design exhibited superior biocompatibility compared to conventional magnetic nanocarriers, particularly for applications requiring direct food contact. The technology has been successfully adapted for LFIA development. Zhai et al. created Fe_3_O_4_-MOF-Pt core–shell composites through electrostatic assembly of PVP-modified Pt nanoparticles with MIL-100(Fe) (Figure 7c) [84]. These composites served as effective immunoprobes for visual detection of carbaryl (CAR) in food samples, combining high sensitivity with rapid detection capabilities. Additionally, microemulsion self-assembly has emerged as an effective “nanoreactor” for property tuning. Zhang et al. developed dual-color magnetic aggregation-induced emission nanoparticles through this approach (Figure 7d) [85]. When applied as signal labels in dual-color lateral flow immunoassay (D-MANP-LFIAs), these materials achieved remarkable detection limits of 0.183 ng/mL for fumonisin B_1_ and 0.017 ng/mL for T-2 toxin, addressing matrix interference challenges in multiplex toxin screening. These three studies demonstrate that magnetic core–shell materials have unlocked the high-throughput potential of LFIAs primarily through two core functions: accelerating sample pretreatment and enabling multi-target integration. This transformation has converted LFIAs from a rapid laboratory-based detection technique into a high-throughput tool capable of supporting the batch screening of food contaminants, thereby providing a viable approach for large-scale sample testing in food enterprises and regulatory authorities.

Despite these advantages, self-assembled core–shell composites face several notable limitations. First, magnetic cores tend to aggregate when encapsulated by degradable polymers or thin inorganic shells. Second, precise control over core size distribution remains technically challenging. Third, the magnetic properties of Fe_3_O_4_ cores are significantly affected by variations in shell characteristics. These factors collectively constrain the stability and reproducibility of such materials, posing challenges for their widespread application in food safety detection [83,86].

#### 2.3.4. Self-Sacrificial Template Method

The self-sacrificial template method represents a strategic synthesis approach that utilizes removable templates—including nanoparticles, polymer microspheres, and metal oxides—to construct precisely controlled material architectures. Following template removal through chemical or physical processes, the resulting functional materials replicate the template’s morphological characteristics, producing hollow, core–shell, or porous structures ideal for developing high-capacity, selective magnetic adsorbents in food safety applications.

Chatzipavlidis demonstrated this approach by selectively dissolving intermediate layers of magnetic nanospheres in ethanol at room temperature, creating magnetic polymer hollow microcontainers with dual magnetic and pH-responsive properties [87]. These structures, when functionalized with target-specific antibodies or aptamers, enable rapid magnetic separation of trace contaminants from food matrices. Huang et al. expanded the template technology from structural regulation to adsorption function enhancement. They advanced the technique using Cu(OH)_2_ as a self-template, converting it into HKUST-1 on Fe_3_O_4_@SiO_2_ cores to produce Fe_3_O_4_@SiO_2_@HKUST-1 nanostructures with exceptional mercury adsorption capacity (264 mg/g) [88]. Further developing this concept, Le et al. synthesized ternary Fe_3_O_4_/CuO@C composites through iron-doped copper-MOF templating, achieving 98.51% ciprofloxacin degradation under visible light and demonstrating outstanding potential for mercury detection in seafood and grains [89]. This design overcame the “single-function” limitation of the previous two studies and enabled the integration of “pollutant degradation” and “pollutant detection”.

While exceptionally versatile for hollow nanostructure fabrication, the sacrificial template method presents notable technical challenges. The template removal process remains complex and risks structural damage, while incomplete removal can compromise material performance—particularly problematic for trace contaminant analysis in foods. These limitations currently necessitate complementary synthesis approaches for optimal results in practical applications.

### 2.4. Practical Applicability, Stability, and Reusability of Different Magnetic Core–Shell Material Synthesis Methods in Large-Scale Food Monitoring

After laboratory-scale preparation of diverse core–shell materials, evaluating the pros and cons of their synthesis methods is crucial—it directly determines feasibility in large-scale food safety monitoring. The in situ synthesis method has a relatively simple operational process and can achieve good target recognition performance through precise shell control (synthesis of MNC@MIPs), but it has higher process complexity and condition control costs in large-scale applications. In contrast, the coprecipitation method (synthesis of CoFe_2_O_4_@ZnO) does not require complex equipment, is easy for mass production, and is more suitable for low-cost large-scale preliminary screening scenarios, requiring only the control of basic parameters. Relying on the characteristics of solvent-free and rapid casting, the physical coating method has greater advantages in large-scale production efficiency; however, the problem of weak interfacial interaction between core and shell (easy to peel off) makes it suitable for only short-term and low-frequency large-scale detection tasks.

From the perspective of the supporting capability of stability and reusability for large-scale monitoring, the in situ synthesis method enables uniform shell growth, and some of its materials exhibit excellent reusability, which can reduce consumable waste in large-scale detection. Although the coprecipitation method has low cost and is easy to use in mass production, its performance degrades significantly after multiple cycles due to poor shell uniformity, requiring frequent material replacement in long-term large-scale monitoring. While the chemical vapor deposition method and self-sacrificial template method have excellent stability or adsorption performance, the former has high equipment costs, and the latter involves complex template removal. Striking a balance between “large-scale” and “economic efficiency” is difficult in both methods, and they are far less suitable for actual food monitoring scenarios than the previous three methods.

## 3. Applications of Magnetic Core–Shell Nanomaterials in Extraction, Enrichment, and Detection of Food Contaminants

### 3.1. Pesticide and Veterinary Drug Residues

The application of pesticides and veterinary drugs has become the norm in agricultural and animal husbandry areas due to the great demand for agriculture and animal husbandry. Large quantities of pesticide and veterinary drug residues, generated in the pursuit of high yields, pose serious hazards to the environment, the food industry, and human health [90,91]. Consequently, developing reliable analytical technologies capable of sensitive, rapid, and accurate multi-residue detection remains critical for monitoring pesticide and veterinary drug residues in animal-derived foods. Equally essential are advanced functional materials that combine efficient enrichment with precise molecular recognition capabilities [92,93,94].

**Figure 8:**
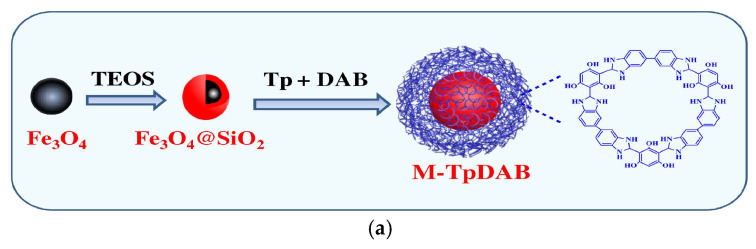

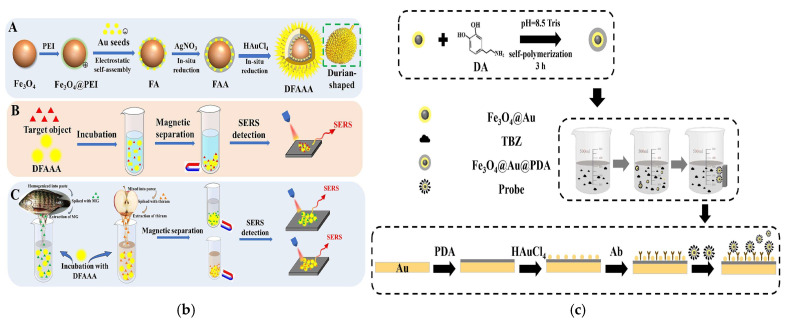
(**a**) The synthesis scheme for M-TpDAB [95]. Copyright Journal of Chromatography A, 2021. (**b**) Schematic illustration of the preparation process for the DFAAA SERS substrate (A). Schematic diagram of the SERS detection process of the DFAAA to the target standard solution (B). Schematic diagram of the destructive (C) and non-destructive [100]. Copyright Food Chemistry, 2024. (**c**) Scheme of the preparation process of Fe_3_O_4_@Au@PDA composite and its application in the detection of TBZ [101]. Copyright Microchemical Journal, 2025.

SPE remains a widely utilized technique to assay pesticide and veterinary drug residues within food matrices, valued for its operational simplicity and reproducibility [95]. However, conventional SPE suffers from lengthy processing times. MSPE has emerged as a superior alternative, offering faster processing, reduced costs, and improved environmental sustainability compared to traditional SPE methods [59,96]. With the goal of improving the detection sensitivity of MSPE targets, Guo et al. developed a new core–shell magnetic covalent organic framework (M-TpDAB) utilizing 3,3′-diaminobenzidine and 1,3,5-triformylphloroglucinol as the starting materials. Combined with HPLC, this MSPE-HPLC approach exhibited excellent detection sensitivity for phenylurea herbicides (PUHs), with LODs ranging from 0.05 to 0.15 ng/mL in water samples and 0.30 to 0.50 ng/mL in beverage samples. Compared with traditional SPE materials, this material can be reused 15 times, significantly reducing the single-use cost. Additionally, this method adopts a solvothermal synthesis approach with simple synthesis steps and does not require expensive detection means, resulting in lower total cost (Figure 8a) [95]. Pan’s team focused on the need to shorten the extraction time of MSPE and created monodisperse magnetic carbon microspheres through phenolic resin coating and subsequent carbonization of Fe_3_O_4_ nanoparticles. These microspheres demonstrated exceptional extraction efficiency for trace triazine herbicides in environmental waters, completing both adsorption and desorption processes within just 2 min [97]. It effectively broke through the technical bottleneck of “lengthy extraction processes” and provided an efficient tool for emergency testing or rapid screening of large batches of samples. Li et al. further expanded the “simultaneous multi-residue analysis” capability of MSPE technology by engineering core–shell magnetic covalent organic framework nanoparticles, Fe_3_O_4_@COFs, for multi-residue analysis. This MSPE adsorbent enabled concurrent quantification of five benzimidazole fungicides in fruits and commercial juices, showing excellent linear responses (R^2^ > 0.99) across 0.01–0.2 μg/mL concentrations [98].

Complementing MSPE, SERS has emerged as a promising technique for rapid pesticide residue analysis. However, conventional SERS substrates face limitations, including lengthy extraction procedures and inadequate selectivity, stability, and reproducibility [99]. The integration of magnetic core–shell materials into SERS systems effectively addresses these challenges while enabling combined enrichment and detection capabilities. Cheshari et al. developed a versatile zero-valent iron-based core–shell support incorporating molecular imprinting technology, which demonstrated nanomolar sensitivity and exceptional selectivity for carbaryl detection [99]. In another advancement, Lv et al. designed a durian-like Fe_3_O_4_@Au@Ag@Au (DFAAA) multilayer core–shell composite (Figure 8b) [100].

Recent advances in functionalized nanomaterials have led to significant improvements in pesticide detection methodologies. It has become the key to breaking through the bottlenecks of traditional detection methods, such as low sensitivity and poor selectivity. Zhang et al., focusing on the core need of improving the sensitivity and specificity of pesticide detection, developed an enhanced surface plasmon resonance (SPR) biosensor incorporating Fe_3_O_4_@Au@PAD core–shell nanoparticles as signal amplifiers, which demonstrated exceptional performance for tebuconazole (TBZ) detection with a remarkably low detection limit of 0.61 ng/mL and a wide linear range of 1–200 ng/mL, while maintaining excellent selectivity against structurally analogous triazole compounds (Figure 8c) [101]. Huang et al., addressing the pain point of low enrichment efficiency and difficulty in recovery of target analytes during the pretreatment of pesticide detection, created pH-responsive MMIPs with core–shell architecture through surface imprinting and radical polymerization techniques, achieving both high adsorption capacity (39.06 mg/g) and selective recognition (imprinting factor = 2.19) for sulfamethoxazole, with the added advantage of pH-controlled binding behavior that enabled efficient analyte recovery (96.2%) in environmental water samples [102]. These innovative approaches exemplify how tailored nanomaterial designs can address critical challenges in pesticide monitoring, offering both enhanced sensitivity and operational flexibility for complex sample matrices.

### 3.2. Antibiotic Residues

Antibiotics are routinely incorporated into animal feed as growth promoters in modern livestock production. Recent studies have detected concerning levels of antibiotic residues in various food products of animal origin, particularly in dairy, poultry, and meat products. Chronic human exposure to these residues through dietary intake has been associated with multiple health risks, including allergic hypersensitivity, the emergence of antimicrobial-resistant pathogens, and potential long-term teratogenic and carcinogenic consequences [103,104]. These findings underscore the critical need for systematic surveillance of antibiotic residues to mitigate food safety hazards and protect consumer health [105].

**Figure 9:**
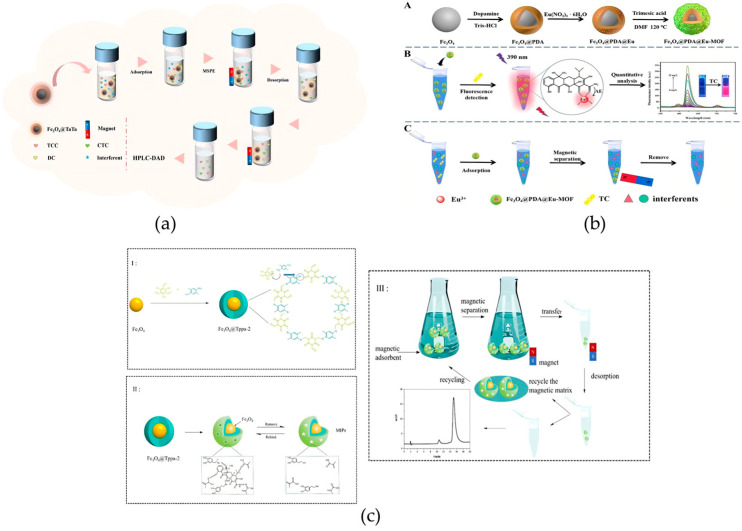
(**a**) Graphical representation of Fe_3_O_4_@TaTa preparation and MSPE procedure for TCs [106]. Copyright Journal of Chromatography A, 2024. (**b**) (A) Schematic illustration of the preparation process for Fe_3_O_4_@PDA@Eu-MOF. (B) and (C) Schematic diagram of detection and isolation of TC using the Fe_3_O_4_@PDA@Eu-MOF [107]. Copyright Chemical Engineering Journal, 2023. (**c**) Schematic illustrations of ultrasonication-based Tppa-2@Fe_3_O_4_ preparation, MIP formation with TC-specific binding cavities, and MIPs acting as magnetic solid-phase extraction sorbents for template purification and enrichment (I-III) [108]. Copyright Food Chemistry, 2022.

In recent years, various advanced enrichment technologies based on magnetic nanomaterials have emerged in the field of antibiotic residue detection research. For example, Sun et al. reported a one-pot in situ self-assembly method to prepare magnetic covalent organic framework composites, Fe_3_O_4_@COFs, with crystalline structures for MSPE of tetracycline (TC) antibiotics in different samples. Experiments showed that Fe_3_O_4_@COFs have excellent enrichment performance and good recyclability for TC, providing a reliable tool for efficient pretreatment of TC residues in high-protein foods such as milk and meat (Figure 9a) [106]. Li et al. also reported a magnetic core–shell material for the detection of TC antibiotics. They constructed a bifunctional core–shell-shell material, Fe_3_O_4_@PDA@Eu-MOF, through layer-by-layer self-assembly technology. Due to its superparamagnetism, distinctive porous framework, and large specific surface area, the material achieves an LOD of 2 μg/L for TC, satisfying the requirement for precise detection of trace TC residues in animal-derived foods (Figure 9b) [107]. Compared with the crystalline COF material developed by the Sun team, the multilayer structure created by the Li team further expands the functional dimension of the material, combines “enrichment efficiency” and “detection sensitivity” more closely, and solves the problem that “trace TC is still difficult to detect accurately after enrichment” in complex matrices. In addition, Han et al. focused on the dual needs for accurate recognition and rapid enrichment, prepared the magnetic covalent organic framework Fe_3_O_4_@Tppa-2 via a room-temperature ultrasonic method, and fabricated core–shell MIPs using precipitation polymerization. These MIPs, serving as MSPE adsorbents integrated with HPLC, were successfully applied in determining TC in real samples such as pork, chicken, and chicken liver. The adsorption equilibrium time is merely 14 min, while the maximum adsorption capacity for TC reaches 87.50 mg/g (Figure 9c) [108].

To date, there have been an increasing number of research reports on the development and application of magnetic core–shell nanomaterials in antibiotic residue analysis. In addition to the aforementioned tetracyclines, they also include aminoglycosides [109], ciprofloxacin [89], fluoroquinolones [65,110], sulfonamides [59,64], etc. The functional integration of the core–shell structure is the key to improving the efficiency and sensitivity of antibiotic residue detection, providing an effective solution for the accurate monitoring of antibiotics in complex food matrices.

### 3.3. Toxin Residues

Mycotoxins are harmful secondary metabolites generated by fungal pathogens, presenting significant contaminant risks to food safety and public health. Therefore, it is essential to create efficient instruments for preventing mycotoxins from accumulating via the food chain. Traditional mycotoxin control strategies often rely on chemical treatments as the core method, but this may lead to residual toxicity and increase the risk of environmental pollution [111,112,113]. Among them, magnetic core–shell nanomaterials, with their distinctive characteristics, including superparamagnetism, large specific surface area, and functionalizable surfaces, have shown significant advantages in the rapid detection of toxins in food and are expected to greatly improve detection sensitivity and efficiency.

**Figure 10:**
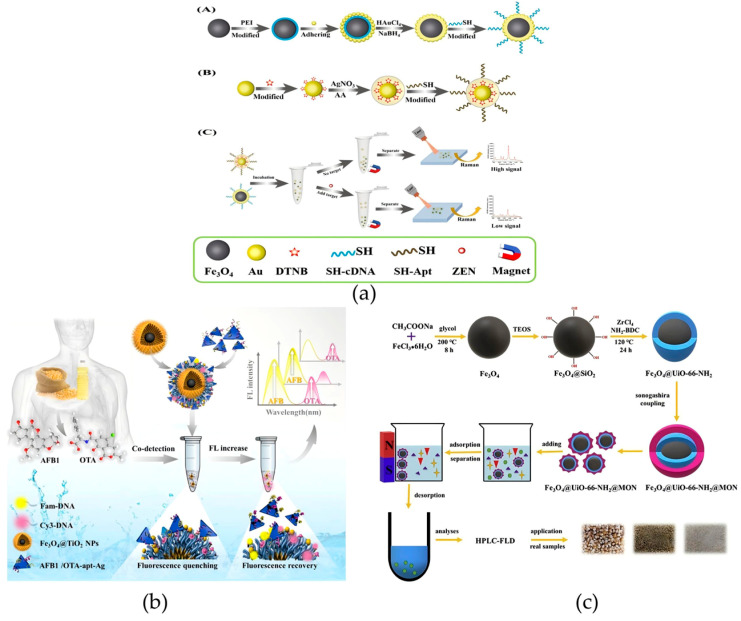
(**a**) Schematic representation of the universal surface-enhanced Raman scattering (SERS) aptasensor platform for trace detection. PEI, polyethyleneimine; AA, ascorbic acid [115]. Copyright *Analytica Chimica Acta,* 2021. (**b**) Schematic illustration of the aptamer-induced Fe_3_O_4_@TiO_2_@AgNPs for AFB_1_ and OTA detection [119]. Copyright Food Chemistry, 2025. (**c**) Synthesis of Fe_3_O_4_@UiO-66-NH_2_@MON as adsorbents for the preconcentration and determination of aflatoxins [120]. Copyright Journal of Hazardous Materials, 2020.

As a mycotoxin produced by fungi belonging to the genus Fusarium, ZEN may contaminate products, thereby endangering the health of animals and humans [114]. In research on ZEN detection, Chen et al. developed a universal SERS aptasensor, which consists of two parts: Fe_3_O_4_@Au as the capture probe and Au@Ag core–shell nanoparticles as the reporter probe. This material solves the problem that traditional SERS is susceptible to matrix interference and exhibits excellent performance in analytical applications for beer and wine (Figure 10a) [115]. In addition, Fu et al. connected β-cyclodextrin (CD) to the surface of magnetic molecularly imprinted polymers (MMIPs) to prepare a novel magnetic molecularly imprinted polymer–cyclodextrin (MMIPs-CD) material, which enabled the rapid and specific adsorption of ZEN. The maximum adsorption capacity of this material is approximately 30 mg/g, with LOQ and LOD of 0.1 ng/kg and 0.3 ng/kg, respectively. Moreover, adsorption equilibrium can be achieved within 20 min, providing a high-performance tool for the rapid concentration of ZEN in grains and feed. The short adsorption equilibrium time and high selectivity make this material an ideal candidate for high-throughput food screening of ZEN in grains—especially for feed factories or grain storage facilities that need to monitor thousands of batches annually [116]. The former improves the detection specificity and anti-interference ability through a SERS aptasensor, while the latter optimizes the adsorption efficiency and selectivity of ZEN via an MMIP-CD composite system, and together they promote the development of the ZEN detection system towards “high efficiency, high sensitivity, and multi-matrix adaptability”.

In addition to ZEN, aflatoxin B_1_ (AFB_1_), as another strong carcinogen, causes serious harm to the health of livestock and humans, so its detection has also attracted much attention [114,117]. Pezeshkpur et al. coupled Fe_3_O_4_@Au nanocomposite adsorbents with MIPs based on molecular imprinting technology and nanotechnology. The magnetic core–shell material prepared by this approach has been successfully employed for the quantification of AFB_1_ in non-alcoholic beer and barley samples, with estimated LOD and LOQ of 6.12 μg/L and 18.6 μg/L, respectively. The adsorption capacity is 8.975 mg/g, and the recovery rate reaches 94.47–97.31% [118]. This material addresses the issues of specific adsorption and quantification of AFB_1_ in specific matrices. Wang et al. constructed a magnetic core–shell material based on AgNTs (energy acceptors) and aptamer-modified Fe_3_O_4_@TiO_2_, which can be used for the concurrent detection of various toxins. The limits of detection for AFB_1_ and ochratoxin A in grain and oil samples are 0.94 ng/mL and 0.54 ng/mL, respectively. This method provides new possibilities for developing aptasensors with high sensitivity and selectivity (Figure 10b) [119]. Li et al. constructed a novel core–shell magnetic microcomposite, Fe_3_O_4_@UiO-66-NH_2_@MON, for AFB_1_ detection in food. Coupled with HPLC, it achieved a detection limit of 0.15–0.87 μg/L. In actual samples, recoveries ranged from 87.3% to 101.8%, demonstrating good application prospects for trace AFB_1_ quantification (Figure 10c) [120]. These studies cover aspects from detection targets and application matrices to detection accuracy, collectively improving the technical system for AFB_1_ detection and providing key technical support for the accurate control of AFB_1_ and related toxins in different food matrices.

**Figure 11:**
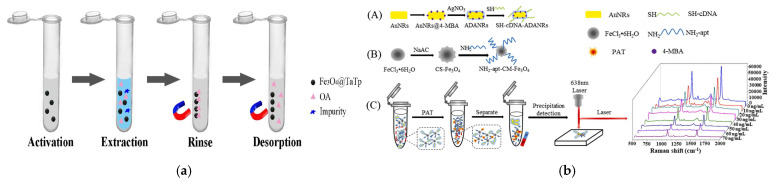
(**a**) The MSPE process with Fe_3_O_4_@TaTp as the material for extracting OA from seawater and shellfish [121]. Copyright Food Chemistry, 2022. (**b**) Preparation procedure of SH-cDNA-ADANRs (A), Preparation procedure of NH_2_-apt-CS-Fe_3_O_4_ (B), SERS detection schematic (C) [122]. Copyright Journal of Food Composition and Analysis, 2023.

In the field of food and environmental toxin detection, besides typical mycotoxins, marine biotoxins (such as okadaic acid) and fruit and vegetable-derived toxins (such as patulin) also pose threats to human health. For these toxins, the application of new functional materials has also shown significant advantages. Cao et al. synthesized core–shell structured magnetic covalent organic frameworks Fe_3_O_4_@TaTp at room temperature using a one-step functionalization method and applied them to the MSPE and LC-MS/MS detection of okadaic acid in seawater and shellfish. Compared with previous reports, this method can not only reduce the amounts of materials used (1 mg for seawater and 5 mg for shellfish) but also greatly shorten the detection time (4 min for seawater and 15 min for shellfish), significantly improving the efficiency and environmental friendliness of toxin detection in seafood (Figure 11a) [121]. Guo et al. prepared a SERS aptasensor by combining chitosan-modified magnetic nanoparticles (CS- Fe_3_O_4_) with gold–silver core–shell structures containing signal molecules (ADANRs). This sensor was used for spectral analysis of apple samples spiked with different contents of patulin, achieving a minimum LOD of 0.0384 ng/mL and a recovery rate of 96.3–108%. The plasmon resonance effect of the noble metal shell and the anti-interference ability of the magnetic core work synergistically, enabling ultrasensitive detection of trace toxins in fruits and vegetables (Figure 11b) [122].

### 3.4. Heavy Metal Ion Residues

Heavy metals, as non-biodegradable and bioaccumulative pollutants, present significant risks to food safety. These contaminants can accumulate in organisms at low concentrations, and certain metal ions exhibit high toxicity even at trace levels, posing serious threats to human health. Effective detection of heavy metals is therefore essential for food safety assurance [123,124,125]. To address the challenge of detecting trace heavy metals in plant-derived food matrices, Morales et al. fabricated a new nanocomposite from multi-walled carbon nanotubes modified with magnetic core–shell Fe_3_O_4_@SiO_2_. It can be effectively applied to vortex-assisted dispersive MSPE of cadmium ions (Cd^2+^) in plant-derived food matrices such as carrots and ginkgo leaves. Its LOD and LOQ are as low as 0.090 μg/L and 0.302 μg/L, respectively, meeting the demand for accurate detection of trace heavy metals [126]. Modheji et al., focusing on the detection of trace arsenic ions in rice, used Fe_3_O_4_@SiO_2_ material, which was encapsulated in an MCM-41-like structure, followed by surface modification with guanidine through an amine linker (GA-MSMP). The guanidine-functionalized mesoporous shell enhances the specific recognition of arsenic ions (As), and the resulting magnetic mesoporous structural material exhibited good adsorption capacity in the detection of trace as in rice [127]. Salman et al., turning their attention to the detection of heavy metals in mineral-rich foods (such as nuts and beans), functionalized the surface of Fe_3_O_4_@SiO_2_ using triethoxysilane as a coupling agent and ethylenediaminetetraacetic acid (EDTA) as a ligand. This material showed superior performance in adsorption and detection of Sc ions, rendering it appropriate for the detection of trace heavy metals in mineral-rich foods [128]. Unlike Morales et al., who focused on improving detection sensitivity, the Long team focused more on enhancing detection efficiency. They devised and developed a novel type of core–shell magnetic Prussian blue-coated Fe_3_O_4_ composite (Fe_3_O_4_@PB) by virtue of the in situ replication and controllable etching properties of Fe_3_O_4_. Adsorption experiments showed that this material has a very fast adsorption rate for Cd^2+^, reaching equilibrium after 4 h with an adsorption rate of 98.78%. This rapid adsorption property significantly shortens pretreatment time for food samples (such as vegetables) and water bodies [129]. MF et al. synthesized composite photocatalytic particles of TiO_2_@CoFe_3_O_4_ with a magnetic core–shell architecture using the coprecipitation method. These particles serve as a new catalyst for the degradation of methylene blue dye and enhance the adsorption efficiency of heavy metal Pb^2+^ ions in aqueous solutions. In Chinese medicinal materials, the maximum adsorption efficiency for Pb^2+^ reached 33.09 mg/g [130]. This study, together with other studies, has jointly formed a heavy metal detection technology network covering multiple types of food and various detection requirements, and comprehensively promotes the development of heavy metal detection technology in food safety testing.

Magnetic core–shell nanomaterials have emerged as indispensable tools for addressing heavy metal contamination in food and water systems. These advanced materials effectively mitigate environmental and health risks posed by persistent, highly toxic metal ions through their unique combination of properties: superparamagnetic behavior, high surface-area-to-volume ratios, and exceptional recyclability. Their operational advantages, including straightforward synthesis, low toxicity, and facile magnetic separation, position them as sustainable solutions for food hazard monitoring, offering both technical efficacy and economic viability [131,132,133].

### 3.5. Non-Compliant Food Additives and Other Hazardous Residues

Food additives are important components of food, as they can endow food with desirable properties (such as preservation, enhancement, and regulation of color and flavor). However, a mounting number of studies have shown that excessive consumption of ultra-processed foods (UPFs) is linked to a rise in non-communicable diseases, along with overweight and obesity [134,135]. These substances are often present in food at low levels with complex matrix interference, placing stringent requirements on the selectivity and enrichment efficiency of detection technologies. Through the synergistic design of “magnetic separation–functional recognition”, magnetic core–shell materials provide an innovative solution for their efficient detection.

**Figure 12:**
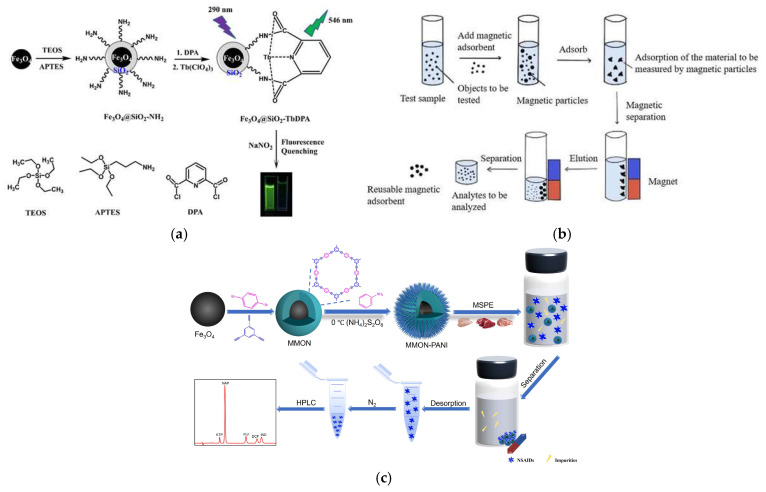
(**a**) Schematic diagram of Fe_3_O_4_@SiO_2_-TbDPA nanoprobe for NO_2_^−^ detection [136]. Copyright Molecules, 2022. (**b**) Flow diagram of MSPE technology for sample pretreatment [137]. Copyright Polymers, 2023. (**c**) Schematic diagram of MMON-PANI fabrication for MSPE of NSAIDs in meat samples [138]. Copyright Molecules, 2022.

Li et al. focused on the need for rapid screening of nitrite (NO^2−^) in food. They prepared an Fe_3_O_4_@SiO_2_-TbDPA nanoprobe based on a magnetic core–shell structure for the detection of NO_2_^−^ in food. The presence of magnetic Fe_3_O_4_ nanoparticles facilitates the effective separation of the material from aqueous solutions, while the probe endows the material with excellent selectivity and sensitivity towards NO_2_^−^ ions. Such high stability, selectivity, and operational simplicity simplify the cumbersome steps of traditional food hazard detection, rendering it appropriate for on-site rapid screening (Figure 12a) [136]. Teng et al. targeted the accurate capture and efficient enrichment of illegal organic additives. They capitalized on the specific binding between sulfonic acid groups and compounds such as melamine and β-agonists. They employed a modified micro-suspension emulsion polymerization method to coat polystyrene on Fe_3_O_4_ magnetic nanobeads; as a result, core–shell magnetic polystyrene microspheres (Fe_3_O_4_@PS) are formed, with Fe_3_O_4_ serving as the core and polystyrene as the shell. The polystyrene shell provides a stable carrier for sulfonic acid groups, enhancing the ability to capture target analytes. Coupled with high-performance liquid chromatography-tandem mass spectrometry (HPLC-MS/MS), this material effectively adsorbs illegal additives such as β-agonists and melamine in food matrices (Figure 12b) [137], offering a new approach for the efficient enrichment and detection of such contaminants. Another research team further expanded the application boundary of magnetic core–shell materials and proposed a new type of material to meet the detection demand for trace non-steroidal anti-inflammatory drug residues in livestock and poultry meat. The core–shell urchin-like polyaniline-modified magnetic microporous organic network (MMON-PANI) prepared by Li et al. integrates the advantages of Fe_3_O_4_, MON, and PANI. The urchin-like structure further expands the contact area with target analytes, improving enrichment efficiency. This material, featuring a considerable specific surface area, quick magnetic responsiveness, and excellent stability, allows for convenient and efficient extraction of trace non-steroidal anti-inflammatory drugs from chicken, beef, and pork samples via the synergistic action of π-π interactions, hydrogen bonding, hydrophobic interactions, and electrostatic interactions (Figure 12c) [138]. The first two studies focus on “rapid screening” and “efficient enrichment–instrument coupling”, while the latter focuses on “trace detection in complex livestock and poultry meat matrices”, forming a comprehensive response to different detection requirements.

Notably, in addition to traditional additives, the contamination of microplastics (MPs) in food is also a matter of concern. Magnetic core–shell materials have demonstrated unique value in the detection and removal of microplastics (emerging contaminants in food). Liu et al. synthesized a MOF material with a magnetic core–shell structure, Fe_3_O_4_@SiO_2_@MIL-53(Al), and applied it to the magnetization and removal of four simulated MPs. Owing to its advantages, such as ease of use and stability, the removal rate of the four MPs reached 54.10–94.17% [139]. Rushdi et al. have carried out innovative research to address the higher demand for synergistic treatment of microplastic composite pollution in food. They fabricated imine-functionalized mesoporous magnetic silica nanoparticles as adsorbents. The imine groups in the mesoporous shell enhance the targeted binding to microplastics, enabling the simultaneous removal of polystyrene microplastics and the organic contaminants adsorbed on them. The synthesized adsorbent exhibited significant reusability over five cycles [140].

To systematically evaluate the performance of magnetic core-shell nanomaterials in food contaminant detection, Table 2 summarizes the key parameters of different materials for the detection of various food contaminants (including pesticides, antibiotics, toxins, heavy metals, and illegal food additives).

**Table 2:** Comparison of magnetic core–shell nanomaterials in food contaminant detection.

Food Contaminants		Magnetic Core–Shell Materials	Detection Methods	Recovery	LOD	Other Key Data	Ref.
Pesticide and veterinary drug residues	CAR	Fe_3_O_4_-MOF-Pt	LFIAs	91.40–102.40% (Vegetables)	0.15 ng/mL	Linear range: 0.25–50 ng/mL	[84]
	PUHs	Fe_3_O_4_@TpDAB	MSPE-HPLC	84.6–105% (Water), 80.3–102% (Beverages)	0.05–0.15 ng/mL (Water), 0.30–0.50 ng/mL (Beverages)	Adsorption (capacities of five PUHs) 10.7–12.1mg/g	[95]
	Sim; Pro	Fe_3_O_4_@COF	MSPE-HPLC	81.44–91.03% (Fruit)	0.01–0.2 μg/mL	Adsorption capacity:387.6 and 448.5 μg/g	[98]
	TBZ	Fe_3_O_4_@Au@PDA	MSPE-SPR	95.8–100.3% (Cucumber), 94.7–102.3% (Corn)	0.61 ng/mL	Linear range: 1–200 ng/mL 3.3-fold signal amplification	[101]
	Thiram	Fe_3_O_4_@Au@Ag@Au (DFAAA)	SERS	89.60–118.34% (Apple)	LOD: 0.13–0.18 ng/cm^2^	enhancement factor: 3.01 × 10^7^	[100]
Antibiotics	TC	Fe_3_O_4_@COFs	MSPE-HPLE	80–120% (Milk, meat)	0.24–0.30 μg/L	Low adsorbent consumption: 5mg Short extraction time: 10 min	[105]
	TC	Fe_3_O_4_@PDA@Eu-MOF	FL	94.7–106.1% (Milk, honey)	2 μg/L	Maximum adsorption capacity: 144.9 mg/g Adsorption equilibrium time: 80 min	[107]
	SAs	Fe_3_O_4_@GC	MSPE-HPLC	77.2–118.0% (Milk)	0.11–0.25 μg/L	Linear range: 1–250 μg/L Enrichment factors: 35.1–39.2 Adsorption equilibrium time: 15 min	[141]
	AGs	Fe_3_O_4_@SiO_2_−NH_2_-MDMIPs	MSPE-HPLC-MS/MS	82.6−114.1% (Milk)	3.6−9.6 μg/kg	Limit of Quantitation: 19 μg/kg (kanamycin sulfate), 25 μg/kg (apramycin sulfate), 32 μg/kg (paromomycin sulfate)	[109]
Toxins	ZEN	Fe_3_O_4_@Au	SERS	96.0% ± 2.2% (Beer) 111.4% ± 3.8% (Wine)	0.001 ng/mL	Adsorption time: 60 min	[115]
	ZEN	MMIP-CD	HPLC-FLD	96.35–98.80%	0.1 ng/kg (Cereals); 3 ng/kg (feed)	Adsorption equilibrium time: 20 min Maximum adsorption capacity: 30 mg/g	[116]
	OTA	Fe_3_O_4_@SiO_2_@UiO-66−NH_2_	MSPE-HPLC	95.83–101.5% (Peanuts)	0.3 μg/kg	Adsorption equilibrium time: 10 min Number of repetitions: three times	[57]
	AFB_1_	Fe_3_O_4_@UiO-66-NH_2_@MON	MSPE-HPLC	87.3–101.8% (Corn, rice, millet)	0.15–0.87 μg/L	Adsorption capacity: 16.3–19.6 mg/g Adsorption time: 10 min	[120]
	OA	Fe_3_O_4_@TaTp	MSPE-LC-MS/MS	96.08–104.82% (Shellfish)	0.04 μg/kg	Pretreatment time: 15 min Adsorbent amount: 5 mg	[121]
Heavy metals	Cd^2+^	MWCNT-Fe_3_O_4_@SiO_2_	VAD-MSPE	96.3–108% (Carrots)	0.090 μg/L	Preconcentration factor: 33.14 Linear range: 0.001–40.0 μg/L	[126]
	As^3+^	GA-MSMP (Fe_3_O_4_@SiO_2_-MCM-41)	MSPE-AAS	98.4–99.8% (Rice)	Not reported	Maximum adsorption capacity: 312 mg/g Number of repetitions: six times	[127]
	Cd^2+^	Fe_3_O_4_@HC	ICP-OES	60–80% (Mussel)	Not reported	Adsorption capacity: 129.87 mg/g	[142]
Illegal additives and others	NO_2_^−^	Fe_3_O_4_@SiO_2_-TbDPA	FL detection	96–108% (Water, meat products)	1.03 μM	Saturation magnetization: 0.075 emu/g	[136]
	NSAIDs	MMON-PANI	MSPE-HPLC-UV	Not reported	0.07–1.7 μg/L	Enrichment factor: 98.6–99.9 Adsorbent consumption: 3 mg (chicken, beef, pork)	[138]
	PVC; PS; PP; PES	Fe_3_O_4_@SiO_2_@MIL−53(Al)	Magnetic Separation-Removal	56.05–97.10% (Liquor)	Not reported	Number of repetitions: five times Maximum adsorption capacity: 10511–44390 mg/g	[139]
	SIB	Fe_3_O_4_@Ag@MIPs	MMIPs-SERS	83.97–91.77% (Tea powder)	1.0 × 10^−9^ M	Number of repetitions: five times, The SERS signal intensity: 1.0 × 10^−9^ M–1.0 × 10^−5^ M	[143]

CAR: carbaryl; PUHs: phenylurea herbicides; Sim: Simazine; Pro: prometryn; TBZ: tebuconazole; TC: tetracycline; Sas: sulfonamides; AGs: aminoglycoside antibiotics; ZEN: zearalenone; OTA: ochratoxin A; AFB_1_: aflatoxin B_1_; OA: okadaic acid; NO_2_^−^: nitrite; NSAIDs: non-steroidal anti-inflammatory drugs; PVC: Polyvinyl Chloride; PS: polystyrene; PP: Polypropylene; PES: Polyethersulfone; SIB: Sibutramine; PDA: polydopamine; YS-Fe_3_O_4_@GC: yolk–shell Fe_3_O_4_@graphitic carbon; Fe_3_O_4_@HC: Fe_3_O_4_-COOH@gamma-CDMOFs; SPR: surface plasmon resonance; HPLC-MS/MS: high-performance liquid chromatography–tandem mass spectrometry; VAD-MSPE: vortex-assisted dispersive MSPE; AAS: Atomic Absorption Spectrometry; ICP-OES: Inductively Coupled Plasma Optical Emission Spectrometer; FL: fluorescence; HPLC-FLD: High-Performance Liquid Chromatography–Fluorescence Detector; ICP-OES: Inductively Coupled Plasma Optical Emission Spectrometer; UV: Ultraviolet; LOD: Limit of Detection; MSPE: magnetic solid-phase extraction; LFIAs: lateral flow immunoassays; SERS: surface-enhanced Raman spectroscopy; MIPs: molecularly imprinted polymers.

## 4. Conclusions and Perspectives

Magnetic core–shell nanomaterials demonstrate significant advantages in the detection of trace contaminants within complex food matrices, such as high magnetic separation efficiency, large specific surface area, and superior adsorption performance, which are attributed to their synergistic core–shell structure. However, several challenges currently hinder their development: (1) weak core–shell interfacial interactions, leading to shell detachment; (2) limited capability for simultaneous enrichment of multiple contaminants; (3) complicated, time-consuming synthesis procedures with low yield, impeding large-scale industrial production; (4) difficulties in controlling shell thickness control; (5) over-reliance on single-category contaminant detection in existing studies.

Future development directions include the following: (1) Exploration of environmentally benign and efficient synthetic approaches for magnetic core–shell nanomaterials. Promising approaches involve using biopolymers as shell components and employing green synthesis techniques assisted by ultrasound, microwave irradiation, or mechanical agitation. (2) Designing multifunctional magnetic core–shell materials capable of simultaneously separating and enriching multiple contaminants, thereby improving the efficiency of sample pretreatment. (3) Optimizing the compatibility of magnetic core–shell materials with high-throughput analytical instruments, such as high-throughput HPLC-MS/MS and automated LFIAs, and integrating them into automated pretreatment-detection systems—this will significantly improve the efficiency of high-throughput food screening, enabling rapid and accurate detection of multi-class contaminants in large-scale food samples such as agricultural products and aquatic foods.

## Figures and Tables

**Table 1:** Comparison of different strategies for magnetic core–shell nanomaterial preparation.

Methods	Advantages	Disadvantages
Coprecipitation method	Environmentally friendly; excellent biocompatible; cost-effective; nanoparticles with narrow size distributions; allowing control over products’ morphology and crystalline phase; easy for mass production	Precise coordination requirement of reaction parameters; poor control over shell thickness/uniformity; uneven distribution of core–shell components; poor compatibility with sensitive cores; poor shell uniformity; obvious performance degradation after cycles
In situ synthesis method	Precise size/thickness control; uniform shell growth; rapid synthesis; suitable for diverse magnetic core–shell composites; uniform shell growth; excellent reusability	Interfacial impurity incorporation; kinetic control challenges; phase compatibility issues; incomplete shell coverage; high cost in large-scale applications; strict control of reaction conditions
Physical coating method	Nondestructive encapsulation; solvent-free processing; large-scale production; solvent-free; high mass production efficiency	Weak interfacial interaction between core and shell; poor uniformity of shell layer; limited control over shell thickness and morphology
Chemical vapor deposition	Solvent-free process; uniform coating formation; high-purity products; controlled thickness; high product purity; excellent stability	High equipment cost; limited to inorganic shells; low production efficiency; complex process control; energy-intensive; high equipment cost; low mass production efficiency
Self-assembly technology	Precise control of shell composition; nanoscale thickness regulation; ordered structural organization; bottom-up fabrication capability; excellent adsorption/stability; controllable structure	Magnetic core aggregation; poor size distribution control; instability of hybrid materials; magnetic sensitivity to shell variations; complex template removal; difficult for large-scale application
Self-sacrificial template method	Versatile template options; precise structure engineering; tunable material properties; hollow/porous architecture fabrication capability; rapid synthesis	Complex template removal; structural damage risks; incomplete-removal issues; requires process optimization; easy magnetic core aggregation; signal attenuation after cycles

## Data Availability

No new data were created or analyzed in this study. Data sharing is not applicable to this article.
